# 

**Published:** 1895-01

**Authors:** 


### INDEX TO VOLUME XLIX.

PAGE

COMMUNICATIONS.

A Basis for Dental Nomenclature, by A. H. Thompson, D.D.S. .......... 583
A History of the Mississippi Valley Dental Society, by E. G. Betty, M.D. .......... 313
Abrasion of the Teeth, by Dr.
American Dental Association.. 590
Antiseptic Dental Surgery, by A. W. Harlan, M.D., D.D S. .......... 595
Carrie M. Stewart, D.D.S. 523
Business Education for Professional Men, by C. D. Blackmarr, D.D.S. .......... 537
Characteristics, by F. E. Battershell, D.D.S. .......... 117
Chairman's Address.. .......... 474
Dental Section of the American Medical Association Meeting at Baltimore, Md., May 6, 1895. "The Value of Different Diagnosis in Dentistry," by Dr. Vida A. Latham, D.D.S. .......... 469
Dental Law—Holland.. .......... 497
Dentistry in Brazil, by T. B.
Discussion on Dr. Case Paper, Continued .......... 597
Mercer, D.D.S. ..........562
Electrical Fusion of Porcelain, by L. E. Custer, B.S., D.D.S. 69
Fiftieth Anniversary of the Discovery of Anæsthesia, by Horace Wells .......... 16
Four Requisites in Development, by M. F. Ault, M.D., D.D.S .......... 573
Gold as a Filling Material, by L. B. Moore .......... 483
Generation and Degeneration of the Tissues of the Mouth, by W. H. Whitslar, M.D., D.D.S. .......... 491
Hygiene of the Mouth, by M. H. Cryer, M.D., D.D.S.. .... 11

PAGE

Harmony, by H. L. Ambler, D.D.S., M.D. .......... 53
Local Anæsthesia .......... 7
Nomenclature, by Grant Molyneaux, D.D.S. .......... 580
Odontalgia, by N. S. Hoff, D.D.S. .......... 105
Ought the Formation of Dental Schools be Limited? by C. W. Stanton, D.D.S. ...... 536
Practical Notes, by L. C. Bryan 593
President's Address .......... 535
Random Thoughts on our Specialty, by A. E. Baldwin, M.D., D.D.S. .......... 8
Report of Special Committee on Dental Nomenclature, by S. H. Guilford, D D.S. 578
Report of Section II, by Louis Ottofy, D.D.S. .......... 589
Resolutions Passed by the American Dental Association at the Last Meeting.. 596
Rigg's Disease; or, "What You Will," by J. E. Craven, D.D.S. .......... 261
Rhisodontrypy, or Hullihen's Operation, by Dr. S. B. Brown, D.D.S. .......... 521
Shedding the Temporary Teeth, by J. Taft .......... 27
Should not the Increase of Dental Schools be Restricted, by Louis Jack, D.D.S. 588
Some Experimental Tests with Sterilizing Agents, by L. P. Bethel, M.D., D.D.S. ... .. 34
Some Incompatibilities, by J. S. Cassidy .......... 608
Some Thoughts on the Teaching of Histology and Anatomy in Dental Colleges, by C. M. Wright, D.D.S.... 78
Separators, by Henry Barnes, D.D.S. .......... 81
Some Peculiarities of Oxyphosphates, by W. V. B. Ames .......... 126

PAGE

The Dentist in His Profession and Among the People, by S. B. Hartman.......... 611
The Present and Future Status of the Dental Practitioner, by Jos. D. Hodgen, D.D.S. 1
The Teeth of our School Children: "What Can be Done to Save them?" by J. C. McCoy, M.D.................. 95
The Use of Sulphuric Acid in the Treatment of Roots for Immediate Filling, by Chas. Welch, D.D.S......... 121
The Reason Why, by Grant Molyneaux, D.D.S... ...... 151
The Hypodermic Use of Cocaine, by H. B. Hinman .. 175
The Origin of Pathological Tendencies, by J. B. Hodgkin, D.D.S.................... 209
The Present Needs in Dentistry, by J. Taft.................. 214
Tumors of the Mouth, by C. G. Darling, M.D................. 365
The Æsthetic Correction of Facial Contours in the Practice of Dental Orthopedia, by C. M. Case, M.D., D.D.S.......................... 540
What a Dentist Saw in Examining Five Hundred Crania, by M. H. Fletcher, M.D., D.D.S...................... 417
Whither are We Drifting? by W. C. Barrett, M.D., D.D.S............................ 587

### MONTHLY DIGEST.

Dental Education, by Mrs. J. M. Walker..................... 44
Operative Dentistry, 102, 132, ........................178, 237, 392
Some Principles Relating to Amalgams, by Mrs. J. M. Walker....................... 448

### REPORT.

Report of Treasurer of World's Columbian Dental Congress in full from March 23, 1891, to July 23, 1895. 564

PAGE

### PROCEEDINGS AND MEETINGS

American Medical Association.................100, 203, 206
Alabama Dental Association—Twenty-Sixth Annual Session.............................. 294
American Dental Association. ................309, 361, 365, 461
East Tennessee Dental Association............................. 310
First District Dental Society of New York—Clinic...... 149
Illinois State Dental Society ..........................253, 310
Louisiana State Dental Society. 102
Mississippi Valley Dental Society.......................... 154
Mississippi Valley Dental Association.........205, 251, 457
National Association of Dental Faculties...........311, 463, 500
National Association of Dental Examiners..............309, 504
National Association of Dental Technics.................. 464
Northern Iowa Dental Society. 364
Ohio State Dental Society.569, 571
Southern Dental Association... 467
Tri-State Meeting. 52, 153, 253, ..............................312, 363
Twelfth International Medical Congress........................ 68

### SELECTIONS.

A New Medical Application of Electricity.................. 513
An Illegal Practitioner sent to Jail..... ......... .............. 563
An Avalanche of Modern Drugs........................... 567
Board of Regent's Request...... 202
Care of the Poor.................. 202
Chemistry as a Servant of Anatomy.................................. 511
Congress of Hygiene............. 512
Delaware's New Medical Law. 456
Double Dislocation of the ~~Superior~~ Maxilla........... 510
Death from Bromide of Ethyl. 401
Eat Slowly........................... 68
Eye-Strain a Cause of Nocturnal Enuresis.................. 202

Inferior

PAGE

Extirpation of the Stomach of a Cat........................... 208
Faith Cure........................... 293
Food for Thought ............... 252
Formaline as a Preserving and Hardening Fluid for Histological Purposes.......... 454
Grief, Emotion and Infection. 99
Guaiacol........................... 456
His Tooth Exploded............ 306
Health Commandments......... 307
How Cocaine Kills.............. 617
Itching of the Mouth ........... 514
New Method of Staining Micro-Organisms in the Blood... 98
Nitroglycerine..................... 509
Neuralgia of the Fifth Nerve—Treatment.................. 508
Phenacetin in Rheumatism.... 402
Pental................................. 452
Properly Classed.................. 515
Phosphorus Poisoning ......... 566
Qualified Practitioners in Belgium............................. 456
Quackery in China.............. 468
Report of the Collective Investigation Committee on Anæsthesia at the Recent Surgical Congress in Berlin............................. 422
Spasmodic Neuralgia of the Face.............................. 403
So Strange........................... 374
Second Childhood................ 293
Statistics............................ 293
Sudden Suppression............. 202
Some of the Causes of Neuralgia............................... 515
The Lay Press and the Medical Profession..................... 236
The Necessity of a Higher Estimation of Tooth and Mouth Hygiene.............. 454
The President of the American Medical Association........ 308
The Health of a Bacillus....... 565
The Teachings of Adversity... 561
Treatment of Cicatrices Pressure and Friction............ 616
Treatment of Phlegmonous Inflammations ............... 569
Ulyptol............................416, 514
What Can a Soldier Do?......... 515

PAGE

COLLEGE COMMENCEMENTS

American Collegs of Dental Surgery........................ 459
Baltimore College of Dental Surgery.......................... 354
Birmingham Dental College... 405
Columbian Dental College...... 409
Columbian University—Dental Department.............. 410
Cleveland University of Medicine and Surgery—Dental Department.................. 411
Chicago College of Dental Surgery ........................ 359
Cincinnati College of Dental Surgery......................... 360
Detroit College of Medicine—Dental Department......... 458
Indiana Dental College.......... 359
Kansas City Dental College.... 412
Meharry Medical College—Dental Department........ 407
Medical Department of University of Denver......... 409
Missouri Dental College........ 358
National University Dental Department.................. 410
New York College of Dentistry ................................ 358
Northwestern College of Dental Surgery.................... 460
Ohio Medical University—Dental Department......... 409
Ohio College of Dental Surgery................................ 355
Pennsylvania College of Dental Surgery................... 355
Philadelphia Dental College 356
Royal College of Dental Surgeons of Ontario............ 406
The Boston Dental College..... 411
University of Buffalo—Dental Department.................. 307
University of California College of Dentistry............ 461
University of Iowa—Dental Department.................. 408
University of Minnesota—Dental Department............. 458
University of Maryland—Dental Department............. 405

PAGE

University of Michigan—Dental Department .......... 412
University of Pennsylvania—Dental Department .......... 459
University of Tennessee—Dental Department .......... 413
Vanderbilt University—Dental Department .......... 407
Western Dental College of Kansas City .......... 404
Western Reserve University—Dental Department .......... 406

### EDITORIAL.

A Morning Star .......... 311
A Return .......... 204
A Well Deserved Honor .......... 414
A Book Reduction .......... 465
A Law to Regulate the Practice of Medicine .......... 517
Bibliographical. 51, 155, 207, 257 .......... 572
Dentistry in Japan .......... 617
Dentist's Relations not Confidential .......... 468
Dr. John S. Billings, U. S. Army .......... 520
Necrology .......... 467
On to Baltimore—By What Way? .......... 260
Personal—Mississippi Valley Dental Society .......... 259
Professional Standing .......... 516
Retirement .......... 204
Removal .......... 519
The American Dentist in Europe .......... 570
The Final Volume of the Index Catalogue .......... 520
The Academy of Stomatology 101
The Sanitarian .......... 154
To the Dentists of the United States .......... 361
To Examine Medical Students .......... 520
Dr. Corydon L. Ford .......... 414
Resolotions Adopted by the Odontological Society of Chicago in Reference to the Death of Dr. J. J. R. Patrick, of Belleville, Ills. 415

### NOTICES.

The Horace Wells Permanent Memorial .......... 256
Medical, Pharmaceutical and Dental Directory .......... 100
Notice .......... 101

### OBITUARY.

Kulp, Dr. William O .......... 102
Brown, Dr. A. W .......... 415
Patrick, Dr. J. J. R .......... 465

### IN MEMORIAM.

Dr. Kulp .......... 103
Dr. J. J. R. Patrick .......... 415

### DIRECTORY OF DENTAL SOCIETIES.

*American Dental Association*—Meets first Tuesday in August, 1895. At Asbury Park, N.J. Pres., J. Y. Crawford, Nashville, Tenn.; Rec. Sec., Geo. H. Cushing, Chicago.

*Southern Dental Association*—Next meeting will be held at Atlanta, Ga., in October, 1895. Pres., H. E. Beach, Clarksville, Tenn. Sec., S. W. Foster, Decatur, Ala.

### STATE DENTAL SOCIETIES.

*Alabama Dental Association*—Meets annually. Next meeting at Mobile, on the second Tuesday of April, 1895. Pres. H. D. Boyd, Troy; Sec. S. W. Foster, Decatur.

*California State Dental Association*—Next meeting will be held in San Francisco, second Tuesday in June, 1895. Pres., L. A. Teague, San Francisco; Rec. Sec., W. Z. King, San Francisco; Cor. Sec., C. E. Post, San Francisco.

*Colorado State Dental Society*—Meets 1st Tuesday in June, 1894, at Glenwood Springs. Pres. P. T. Smith, Denver; Sec., Sarah May Townsend, Denver.

*Connecticut State Dental Society*—Next meeting will be held in Hartford, third Tuesday in May, 1895. Pres. Chas. P. Graham, Middletown; Rec. Sec. Geo. L. Parmele, Hartford.

*Dental Society of the State of Maryland and District of Columbia*—Meets annually. Next meeting at Washington City, on the second Tuesday in October, 1893. Pres. B. F. Coy, of Baltimore: Sec. M. W. Foster, Baltimore.

*Florida State Dental Association*—Meets annually. Next meeting at Tampa, 1st Tuesday in May, 1895. Pres, W. A. McQuaig, Lake City; Cor. Sec. S. W. Allen, Tampa.

*Georgia State Dental Society*—Meets at Indian Spring, in 1895. Exact time to be decided later; Pres. W. W Hill, Washington; Rec. Sec. S. H. McKee, Americus; Cor. Sec. O. H. McDonald, Atlanta.

*Illinois State Dental Society*—The next meeting will be held at Galesburg, Ill., May 14 to 17, 1895. Pres. J. W. Cormany, Mt Carroll., Sec. Louis Ottofy, Chicago.

*Indiana State Dental Society*—Will meet at Detroit, Mich., on the third Tuesday of June, 1895. Pres. D. S. Harrold, Elwood; Secretary, G. E. Hunt, Indianapolis.

*Iowa State Dental Society*—Meets annually. Next meeting at Iowa City, May, 7th to 10th, inclusive, 1895. Pres. J. S. Kulp, Muscatine; Rec. Sec. F. T. Breene, Iowa City.

*Kentucky State Dental Association*—Meets annually. Next meeting in Louisville, Ky., 3rd Tuesday in June, 1894. Pres., J. H. Letcher, Danville; Sec., J. H. Baldwin, Louisville.

*Kansas State Dental Association*—Pres. C. B. Reed, Topeka; Sec., W. W. West, Topeka. Next meeting will be held at Topeka, second Monday in May, 1895.

*Maine Dental Society*—Will meet on the third Tuesday in July, 1895, in Bangor. Pres. T E. Tibbetts, Rockland; Sec. F. A. Knowlton, Fairfield.

*Maryland State Dental Association*—Next annual meeting will be held (place not definitely known yet,) in April, 1895. Pres., Chas. C. Harris, Baltimore; Sec, W. W. Dunbracco, Baltimore.

*Massachusetts Dental Society*—Meets annually in Boston, in June. Pres. Josiah W. Ball, Boston; Rec. Sec., E. O. Kinsman, Cambridge.

*Michigan State Dental Association*—Meets annually. Next meeting at Detroit, on the 3rd., Tuesday of June, 1895. Pres., W. P. Morgan, Saginaw; Sec., J. Ward House, Grand Rapids; Treas. Geo. H. Mosher, Jackson.

*Mississippi Dental Association*—Next meeting the first Wednesday in April, 1895, at Jackson, Miss. Pres. W. E. Walker, Pass Christian; Sec. T. C. West, Natchez.

*Minnesota State Society*—Pres. C. H. Stearns, Zumbrota; Sec., C. W. Jones, St. Paul. Will meet in Minneapolis, first week, in September, 1894.

*Missouri Dental Association* Meets at Bertle Springs, Mo., second Tuesday in July, 1895. Pres., J. T. Fry, Moberly; Sec., W. W. Carter, Sedalia.

*Nebraska State Dental Society.*—Next annual meeting will be held at Norfolk, third Tuesday in May, 1895. Pres., H. W. Shriver, Omaha; Cor. Sec., O. M. Huestis, Nebraska City.

*New Jersey State Dental Society*—Meets annually The next meeting will be held at Asbury Park, on the 2 l of August, 1895. Pres. C. W. F. Holbrook, Newark; Sec. C. A. Meeker, Newark.

*New Hampshire Dental Society*—Meets Annually. Next meeting will be held in Concord, N. H.; time to be decided later. Pres. W. R. Blackstone, Manchester; Sec. Burton C. Russell, Keene.

*North Carolina State Dental Society*—Meets in Salisbury, on the second Tuesday in May, 1895. Pres H. H. Harper, Kinston; Sec., J. E. Wyche, Greensboro.

*North Dakota State Dental Society*—The next meeting will be held at Fargo, May, 1895. Pres. E. M. Pierce, Hillsboro; Sec. C. L. Rose, Fargo.

*Ohio State Dental Society*—Meets annually. There will be a meeting of this Society with the Indiana and Michigan Societies at Detroit, Mich. on the 3rd Tuesday of June, 1895. Pres. Wm. H. Todd, Columbus; Sec. L. P. Bethel, Kent.

*Pennsylvania State Dental Society*—Will hold its next annual meeting at Eagle's Mere, Pennsylvania, July 9th, 1895. Pres., Dr. J. A. Libbey, Pittsburgh; Cor. Sec., H. E. Roberts, Philadelphia.

*Rhode Island Dental Association*—Pres., C. M. Noble, Providence; Sec., H. W. Gillett, Newport, Meets quarterly, second Tuesday in July, October, January and April. Annual Meeting second Tuesday in July, at Newport.

*South Carolina State Dental Association*—Meets annually. Pres. J. C. Orland, Spartanburg; Cor. Sec. C. B. Colson, Charleston. Next meeting at Spartanburg, October, 1895.

*Tennessee Dental Association*—Meets annually. Next meeting at Knoxville, July 9, 1895. Pres. W. H. Richards, Knoxville: Rec Sec. W. P. Houston, Lawrenceburg; Cor. Sec. W. J. Morrison, Nashville.

*The Dental Society of the State of New York*—Meets annually on the second Wednesday in May. Next session at Albany, 8th and 9th of May, 1895. Pres. F. T. Van Woert, Brooklyn; Sec. C. S. Butler, Buffalo; Cor. Sec. R. Ottolengui, New York.

*Texas State Dental Association*—Meets in San Antonia, third Tuesday in **May**, 1894. Pres. A. A. Beville, Waco; Sec. Chas. B. Lewis, Ennis.

*Vermont State Dental Society*—Meets annually. Next meeting will be held at Brandon, Vt., third Wednesday in March, 1895. Pres. W. H. Wright, Brandon; Sec. Thos. Mound, Rutland.

*Virginia State Dental Association*—Will meet in 1895. Exact time and place to be decided later. Pres. H. W. Campbell, Suffolk; Sec. J. Hall Moore, Richmond.

*Washington State Dental Society*—Meets Annually. Next meeting will be held at Seattle, in the middle of May, 1895. Pres. B. S. Scott, Ellenburg; Sec., R. B. Gentle, Olympia.

*Wisconsin State Dental Society*—Meets annually. Next meeting will be held at Madison, Wis., the third Tuesday in July, 1895. Pres., Chas. C. Chittenden, Madison; Sec., C. A. Southwell, Milwaukee.

*West Virginia State Society*.—The annual meeting will be held at Parkersburg, on the first Wednesday of October, 1894. Pres. J. N. Mahan, Charleston; Sec. Geo. I. Keener, Morgantown.

### LOCAL SOCIETIES.

*American Academy of Dental Science*—Meets annually in Boston, Mass., on the first Wednesday in October. Pres. J. H. Batchelder; Rec. Sec. William Y. Allen.

*Buffalo Dental Association*—Meets last Monday evening of each month. Annual meeting in June. Pres. F. H. Boswell; Sec. J. A. Stackhouse.

*Brooklyn Dental Society and Second District Society United*—Meets quarterly—Pres. T. H. Holly; Rec. Sec. A. N. Russell, Brooklyn.

*Central Dental Association of Northern New Jersey*—Meets bi-monthly; and annually. The next annual meeting will be held about the middle of February, 1895. Pres. Thomas Moore, Paterson; Sec. Wm. L. Fish, Newark.

*Chicago Dental Society*—Meets monthly. Pres. J. H. Woolley; Sec. A. H. Peck; Cor. Sec H. A. Costner.

*Chicago Dental Club*—Meets on the fourth Monday of each month. Pres. W. H. Taggart; Sec. E. L. Clifford.

*Cleveland Dental Society*.—Meets the first Monday of each month. Pres. S. B. Dewey; Sec. Martha J. Robinson.

*Connecticut Valley Dental Society*—Meets annually. Next meeting in Oct. 1895, time and place to be decided later. Pres. C. C. Barker, Meriden, Conn.; Sec. Geo. A. Maxfield, Holyoke, Mass.

*Detroit Dental Society*—Meets monthly, Pres. Geo. L. Field, Sec. A. W. Diack.

*Eighth District Dental Society, N. Y.*—Next annual meeting April 24th, 1894, in Buffalo. Pres. F. J. Burkhart, Batavia; Sec., W. V. Grove, Buffalo.

*East Tennessee Dental Association*—Meets annually. Next meeting will be held at Harriman, second Tuesday in June 1895. Pres. F. A. Shotwell, Rogersville; Sec. and Treas. Geo. C. Brause, Harriman.

*Fifth District Dental Society of N. Y.*—Meets semi-annually. Next annual meeting will be held in Utica, 2d Tuesday in April, 1895. Pres. Frank Jones, Syracuse; Cor. Sec. A. R. Cooke, Syracuse.

*First District Dental Society of the State of New York*—Meets annually. The next meeting will be held on the second Tuesday of April 1895. Pres., Victor Hugo Jackson, New York City; Sec., Benj. C. Nash, New York City.

*First District Dental Society of Illinois*—Next meeting will be held in Canton, second Tuesday in September, 1895. Pres. W. A. Johnston, Peoria; Sec. W. O. Butter, La Harpe.

*Knoxville City Dental Society*—Meets monthly. President, J. T. Cazier; Sec. A. J. Cottrell.

*Minnesota State Dental Society*—Annual Meeting will be held in St. Paul, 2nd Wednesday in Sept., 1895. Pres. C. H. Robinsin, Wabasha; Sec. W. L. Cruttenden, Northfield.

*Mississippi Valley Dental Society*—Meets annually at Cincinnati. Next meeting March, 1895. Pres., J. Taft, Cincinnati; Rec. Sec. H. T. Smith, Cincinnati; Cor. Sec. H. C. Matlack, Cincinnati, O.

*New England Dental Society*—Meets annually. Next meeting 2nd Thursday in Nov., 1894, in Boston Mass. Pres. J. F. Adams, Worcester, N. H.; Sec. E. O. Kinsman, Cambridge, Mass.

*Northern Illinois Dental Society*.—Meets annually. Next meeting at Aurora, October 17th, 1894. Pres. E. R. Warner, Chicago; Sec. J. W. Cormany, Mt. Carroll.

*Northern Ohio Dental Association*—Meets annually. Next meeting at Put-in-Bay, O., June 19th, 1895. Pres. Henry Barnes, Cleveland; Rec. Sec. F. N. Knowlton, Akron; Cor. Sec. L. L. Barber, Toledo.

*Northwestern Dental Association* (embraces portions of Dakota, Minnesota and Manitoba) meets annually. Next meeting at Detroit, Dak. on the fourth Tuesday in July, 1892. Pres. J. W. Cloes, Jamestown, Dak. Sec. S. J. Hill, Fargo, Dak.

*Odontological Society of Pennsylvania*—Meets on the first Saturday evening of each month, at 8 o'clock p. m., at 13th and Arch sts; Pres. Jas. Truman, Rec. Sec. A W. Deane.

*Odontological Society of New York City*—Meets the third Tuesday of each month. Pres. Wm. Jarvie; Rec. Sec. E. T. Payne.

*Odontological Society of Cincinnati*—Meets monthly. Pres. G. Molyneaux; Sec. H. C. Matlack.

*Odontological Society of Chicago*—Meets monthly. Pres. W. V. B. Ames; Sec. and Treas., Louis Ottofy.

*Pennsylvania Association of Dental Surgeons*—Meets second Tuesday of each month. Pres. H. E. Roberts; Cor. Sec. Theo. F. Chupein.

*Pittsburg Dental Society*—Meets the last Tuesday Evening of each month. Pres. J. B. Williams, Homestead; Sec. N. L. Reinecke.

*San Francisco Dental Society*—Meets the second Monday evening of each month. Annual Meeting in October. Pres. C. E. Post; Sec. G. N. Van Orden; Cor. Sec. H. G. Richards.

*Sixth District Dental Society of N. Y.*—Meets semi-annually. The annual meeting will be held at Binghamton, the first week in May, 1895. Pres., M. H. Fish, New Berlin; Sec., F. W. McCall, Binghamton.

*Seventh District Dental Society of Ohio*—Meets annually. Next meeting in May, 1894. Pres., O. N. Heise, Cincinnati; Sec., J. R. Callahan, Cincinnati.

*Southern Illinois Dental Society*—Meets annually. Next annual meeting will be held at Nashville, Ill., on the 3d Tuesday in October, 1894. Pres., L. B. Torrence, Chester; Sec., G. W. Entsminger, Carbondale.

*Southern Minnesota Dental Society*—Next meeting in Mankato, third Tuesday in April, 1895. Pres. S. Bond, Onoka; Sec. F. B. Kremer, Minneapolis.

*St. Louis Dental Society*—Meets first and third Tuesday in each month. Pres. J. B. Newby; Cor. Sec. John G Harper; Rec. Sec. F. F. Fletcher.

*The Isaac Knapp Dental Coterie of Fort Wayne, Ind.*—Meets the first Thursday of each month. Pres., W. W. Mungen; Sec., M. A. Mason.

*The Minneapolis Dental Society Monthly*—Pres. E. F. Clark, Sec. E. J. Morrison.

*The Tacoma Dental Society*—Meets monthly. Pres. M. C. Burns; Sec. W. E. Burkhart.

*Washington City Dental Society*—Meets 3d Tuesday of each month. Pres. J. H. P. Benson; Rec. Sec. J. Roland Walton.

To Dr. Bodecker the profession is under an obligation in this matter, which they can in part at least repay by conscientious study and thorough understanding of all the subjects that are embraced in the work.

The work is obtainable through any bookseller, or of any of the dental depots. Price $5.00.

### NOTICES.

### Tri-State Meeting.

The joint meeting of the Dental Societies of Ohio, Michigan and Indiana will occur June 18-19-20, 1895, at Detroit, Michigan. The dental department of the Detroit College of Medicine and Surgery has been secured for the sessions. Michigan has generously invited her sister States to share her hospitality and be her guests on this occasion. The program contemplates four literary sessions, two half days of clinics, and one half day of "hurrah." This latter will come in form of an excursion thirty-two miles up the Detroit River to the St. Clair Flats, where we will dine at one of the club houses built on piles in the middle of Lake St. Clair. Special hotel and railroad rates are assured and will be announced later. The railroad fare will be at least as low as one and one-third fare. All reputable dentists in the three States are cordially invited to attend.

EXECUTIVE COMMITTEE.

### CONTENTS.

COMMUNICATIONS.

The Present and Future Status of the Dental Practitioner .......... 1

Local Anæsthesia .......... 7

Random Thoughts on our Specialty.......... 8

Hygiene of the Mouth .......... 11

Fiftieth Anniversary of the Discovery of Anæsthesia by Horace Wells 16

Shedding the Temporary Teeth .......... 27

Some Experimental Tests with Sterilizing Agents .......... 34

Monthly Digest .......... 44

EDITORIAL.

Bibliographical .......... 51

NOTICES.

Tri-State Meeting .......... 52

---

### THE DENTAL REGISTER,

A MONTHLY MAGAZINE DEVOTED TO THE INTERESTS OF THE

**DENTAL PROFESSION.**

*Terms Reduced to* $2.00 *Per Year. Single Copies,* 25 *Cents.*

**EDITED BY PROF. J. TAFT, D.D.S.**

——AND——

**Published by SAM'L A. CROCKER & CO., Ohio Dental and Surgical Depot.**

The volume commences in January and closes in December.

The REGISTER being issued monthly is always fresh, therefore Indispensable to the practitioner who desires to keep posted up with the new inventions and improvements which are daily developed. Neither expense nor effort will be spared to give value and variety to its contents. Articles will be illustrated with well executed engravings whenever they will add to the interest or assist to explanation.

Its extensive circulation and frequency of issue make it one of the BEST DENTAL ADVERTISING MEDIUMS in the United States.

All subscriptions must begin with January or July.

Local or special notices, five lines or less, will be inserted for $3 each insertion ; over five lines, 50 cts. per line.

All advertising bills payable in advance, or at furthest, after the issue of the first number containing the advertisement. All transient advertisements to be paid for in advance.

☞CONTRIBUTIONS SOLICITED.

Exclusively Editorial Communications should be addressed to the Editor.

The latest number of the REGISTER may be ordered with or without other goods any time, and all letters should be addressed to

